# Effects of knee flexor submaximal isometric contraction until exhaustion on semitendinosus and biceps femoris long head shear modulus in healthy individuals

**DOI:** 10.1038/s41598-020-73433-1

**Published:** 2020-10-02

**Authors:** Bruno Mendes, Telmo Firmino, Raúl Oliveira, Tiago Neto, Carlos Cruz-Montecinos, Mauricio Cerda, José P. Correia, João R. Vaz, Sandro R. Freitas

**Affiliations:** 1 Desporto e Saúde, CIPER, Faculdade de Motricidade Humana, Universidade de Lisboa, Estrada da Costa, 1499-002 Cruz Quebrada Dafundo, Portugal; 2 Sport Lisboa e Benfica, Human Performance Department-Health Performance, Av. Eusébio da Silva Ferreira, 1500-313 Lisboa, Portugal; 3 Escola Superior de Saúde do Alcoitão, Rua Conde Barão, 2649-506 Alcabideche, Cascais, Portugal; 4 Departament of Physiotherapy, LUNEX International University of Health, Exercise and Sports, Differdange, Luxembourg; 5 Department of Physical Therapy, Laboratory of Clinical Biomechanics, Faculty of Medicine, University of Chile, Santiago, Chile; 6 Anatomy and Developmental Biology Program, Institute of Biomedical Sciences (ICBM), Faculty of Medicine, Universidad de Chile, Santiago, Chile; 7 Biomedical Neuroscience Institute, Independencia, 1027 Santiago, Chile

**Keywords:** Ultrasonography, Skeletal muscle

## Abstract

This study examined whether a knee flexor isometric contraction at 20% of maximal voluntary isometric contraction until exhaustion would alter the biceps femoris long head (BFlh) and semitendinosus (ST) active stiffness, assessed using ultrasound-based shear wave elastography. Twelve healthy individuals participated in 2 sessions separated by 7 days. Time to exhaustion was similar in both sessions (day 1: 443.8 ± 192.5 s; day 2: 474.6 ± 131.7 s; p = 0.323). At the start of the fatigue task, the ST showed greater active stiffness than the BFlh (p < 0.001), with no differences between days (p = 0.08). The ST active stiffness then decreased from 40% of the task time to exhaustion (− 2.2 to − 13.3%, p = 0.027) until the end of the task (− 16.1 to − 22.9%, p = 0.012), while no significant changes were noted in the BFlh (p = 0.771). Immediately after the fatigue task, a decrease in active stiffness was observed in the ST (− 11.8 to − 17.8%, p < 0.001), but not in the BFlh (p = 0.551). Results were consistent between the 2 testing sessions (p = 0.07–0.959). The present results indicate that fatigue alters the hamstring active stiffness pattern.

## Introduction

Humans can use different neuromuscular strategies to perform a given body posture or joint motion^1–3^. A particular case is the so-called ‘load sharing’^4–6^. This refers to the distribution of force amongst synergistic muscles to generate joint torque. Classical approaches have considered that the individual muscle contribution is mostly dependent on the muscles’ physiological mechanical advantages (e.g. moment arm and cross sectional area)^7,8^; but other factors are also thought to play an important role in determining load sharing, such as fatigue^5,9^.

In the presence of fatigue, synergistic load sharing is generally assumed to be governed by an efficiency principle where the total energetic cost to maintain workload is minimized^5,10^. This means that less fatigable muscles (i.e. with a higher proportion of slow-twitch fibers) should play a greater role in maintaining workload when fatigue is installed. Considering the muscle force-stiffness (linear) relationship^11,12^, recent studies have examined the muscle active stiffness (i.e. during contraction) using ultrasound shear wave elastography (SWE) to determine if fatiguing exercises could alter the load sharing pattern^13–16^. Most of these studies were performed in the quadriceps muscles; overall findings suggest that either a consistent or variable inter-individual load sharing response could be related to the type of fatiguing protocols. For instance, submaximal and constant fatiguing exercises have produced a highly variable quadriceps load sharing pattern^13,15^, while fatiguing protocols with intermittent contractions and higher contraction intensities may induce a systematic load sharing pattern^14,16^. It should be noted that the quadriceps muscle heads converge into a single tendon, whereas the fiber type composition in certain quadriceps muscles (e.g. *vastus lateralis*) vary greatly between individuals^17^. Thus, such a premise should be tested in synergist muscle complexes with multiple tendon insertions, where biomechanical properties vary between muscle components. In addition, it is also important to test the load sharing at different time moments to determine whether responses to fatigue are consistent over time.

The hamstring muscle group plays an important role in human locomotion. For instance, it is the main actuator in the propulsion phase during walking^18^ and sprinting^19^. This muscle group exhibits several characteristics that make it unique and highly complex: it acts as both a knee flexor and hip extensor; and presents heterogeneous morphology, fascicle architecture, and fiber type content among the muscle components^20–22^. It also shows greater fatigability compared to the quadriceps^23^. In terms of physiological factors related to the load sharing, a similar pattern seems to exist in the hamstrings at the neural^24^, metabolic^25,26^, and stiffness^27^ level during knee flexion tasks. The semitendinosus (ST) has been shown to display greater neural, metabolic, and stiffness responses during a knee flexion task as compared to the remaining hamstring muscles, suggesting the load sharing strategy favours a greater ST workload. However, as physiological and mechanical differences exist between the hamstring heads^20–22^, it is possible that responses to fatigue could also diverge between the muscular components in terms of active stiffness. Compared to the ST, the biceps femoris long head (BFlh) presents different functional (i.e. ST performs tibial internal rotation while BFlh performs tibial external rotation^28^) and architectural (i.e. fusiform ST vs. pennate BFlh^20^) properties. In addition, the ST presents greater metabolic (i.e. greater metabolic activity)^25,29^, and neural^30^ responses to fatiguing knee flexor tasks. Thus, it is possible that a different muscle active stiffness response occurs with fatigue.

This study aimed to examine the BFlh and ST active stiffness response during and immediately after a knee flexor isometric submaximal contraction until exhaustion. Additionally, it was examined if responses to fatigue would be consistent between two separate testing sessions. Under the premise that with fatigue, synergistic muscles adopt the most efficient strategy to sustain the mechanical output and increase task endurance, and assuming the possibility that fast-twitch fiber content is higher in ST compared to BFlh^22,31^, we hypothesized that the neuromuscular system would delay the task failure by decreasing the ST active stiffness. Additionally, we hypothesized that the individual responses would be consistent between sessions.

## Material and methods

### Participants

Twelve healthy and physically active male adults (age: 23.3 ± 2.1 years; height: 1.77 ± 0.08 m; body mass: 74.4 ± 9.8 kg) with no history of lower limb injuries in the past 2 years volunteered to participate in this study. Participants were instructed not to perform strength or flexibility training at least 72 h prior to the testing sessions. Informed consent was obtained from participants before the data collection. This study was approved by the local ethics committee (Ethics Council of Faculty of Human Kinetics—CEFMH—approval number: 21/2016), and the procedures were conducted according to the principles expressed in the Declaration of Helsinki.

### Equipment and variables

#### Dynamometry

**Figure 1:**
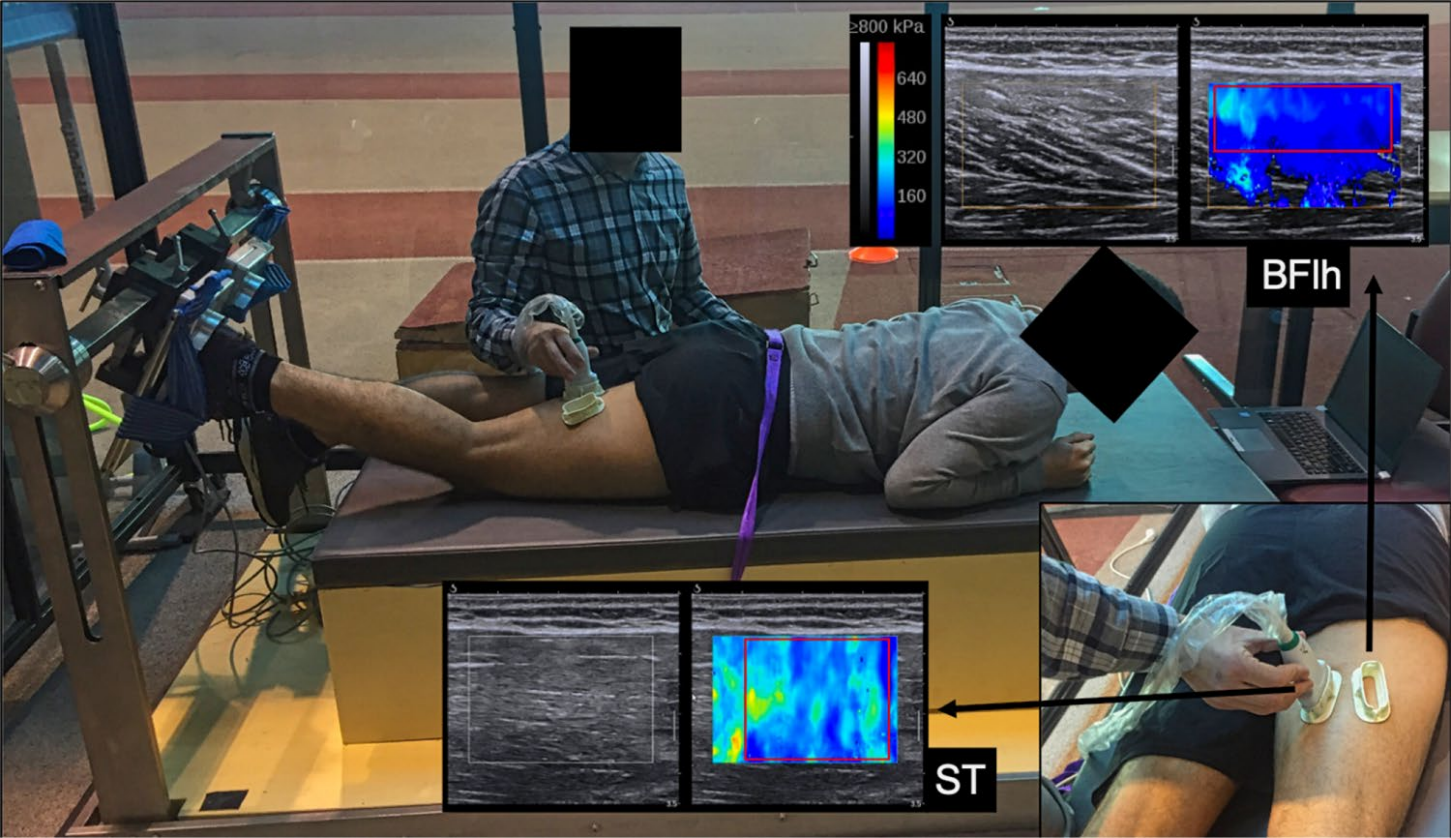
Experimental setup used to assess the localized active stiffness (i.e. shear modulus) of biceps femoris long head (BFlh) and semitendinosus (ST) during a knee flexion isometric contraction at 20% of maximal voluntary isometric contraction, with visual feedback of knee torque production during the assessments. The b-mode (left) and shear wave elastography mode (right) sonograms from one representative study individual are also shown. In the elastograms, the area of the region of interest where the analysis was performed is drawn in red. This image was performed for setup demonstration; thus, to ensure image clarity, the ultrasound scanners or the second examiner are not shown in the picture. Elastograms images were obtained from the Aixplorer's software, v10. URL: https://www.supersonicimagine.com/Aixplorer-R/Aixplorer.

The knee flexor torque was measured at a sampling rate of 1 kHz using a custom-made equipment (Fig. 1). The individuals were placed in the prone position, with the hips in neutral position and the tested knee flexed at 30° (0° = full extension) with the tibia in neutral position. This position allows for the assessment of muscle stiffness with minimal passive tension. The foot of the tested limb was fixed in a foot holder which contained a force transducer (Model STC, Vishay Precision, Malvern, PA, USA; Fig. 1) at the heel level to collect the linear force perpendicular to the leg orientation and with the ankle at 90°. Force data was amplified (Model UA73.202, Sensor Techniques, Cowbridge, UK), digitally converted (USB-230 Series, Measurement Computing Corporation Norton, MA, USA), recorded using the DAQami software (v4.1, Measurement Computing Corporation, Norton, MA, USA), and multiplied by the perpendicular distance between the force transducer center and the femoral lateral condyle in order to estimate the knee torque. Visual feedback of force production was provided to individuals during the assessments.

#### Ultrasound-based shear wave elastography

The muscles’ active stiffness was estimated by shear modulus quantification using two ultrasound scanners (Aixplorer, v10; Supersonic Imagine, Aix-en-Provence, France) in SWE mode (musculoskeletal preset, penetrate mode, smoothing level 5, persistence off; scale: 0–800 kPa). Each scanner was coupled with a linear transducer array (SL10-2, 2–10 MHz, Vermon, Tours, France). The push frequency that generated the elastogram window was set automatically by the ultrasound equipment to approximately 1 Hz (range: 0.8–1.4 Hz), depending on the elastogram window size and position (i.e. frequency decreases for larger windows and at higher depths). The ultrasound probes were placed at 55% of the distal-to-proximal femur length. This site was chosen since it was previously observed that this location corresponded to a larger cross-sectional area of both muscles, where fascicles could be well visualized during contraction without considerable presence of intramuscular aponeuroses^27^. To increase the measurement consistency across repeated trials and ensure that the muscle shear moduli were measured in the same site, a plastic cast was fixed to the skin on the cutaneous projection of each muscular region of interest using bi-adhesive tape (Fig. 1). The casts were orientated according to the fascicles’ direction during a submaximal contraction in each muscle. Two examiners were trained (~ 25 h training) to perform the SWE assessment and were unaware of the study purpose. Examiners held the ultrasound probes during the shear modulus assessment with minimal pressure, with care to not cross the ultrasound probes’ observation planes so as not to produce echographic noise. Measurements were taken at a constant room temperature of 25°. Clip videos with both B-mode and elastogram windows were recorded during the tests. In addition, the assessment of hamstring muscles active stiffness component during submaximal isometric contractions was recently demonstrated to be reliable^27^.

The SWE technique has been previously described in detail^32,33^. Briefly, the velocity ($$V_{s}$$) of shear waves generated by a pushing beam within the muscle are measured by a time-of-flight algorithm in each pixel of the elastogram map in ultrafast ultrasound sequences. The shear modulus ($$\mu$$) is then calculated using $$V_{s} $$ as follows: $$\mu = \rho V_{s}^{2} { }$$, where ρ is the muscle mass density (1000 kg/m^3^). It should be noted that shear wave velocity has been interpreted as a proxy of both the active forces derived from contractile machinery (when the force-stiffness relationship is preserved^11^) and tissue intrinsic stiffness^9,34,35^.

### Protocol

Participants were tested in two sessions, separated by 7 days, at the same time of the day. Six participants (randomly chosen) had their right limb tested, while the left limb was tested in the other six. The session started with a standardized warm-up (i.e. 20 submaximal and 5 maximal knee flexions), followed by two 5-s MVIC trials with 1-min recovery between trials. After a 5-min rest, the shear modulus of each muscle was assessed at 20% MVIC during a 15-s knee flexion isometric contraction. The two muscles were assessed simultaneously by the two probes during each contraction. One ultrasound transducer always measured the ST and the other measured the BFlh. Two trials were performed, with a 30-s rest between trials. Afterwards, the individuals performed the sustained submaximal isometric knee flexion at 20% of MVIC until exhaustion (i.e. when the force produced decreased by 5% from the required target contraction intensity for more than 5 s, or the individual reported being unable to continue the task). Visual assessment of torque production, with a clear indication of the torque corresponding to 20% of MVIC, was given to individuals. Verbal encouragement was always given to individuals during the fatigue task to maintain the torque production at the 20% of MVIC visual line. During the task, the two ultrasound probes were placed over the BFlh and ST continuously until the end of the task. Since the maximum recording time of clip videos with SWE-mode was 60-s, successive video clips were recorded until the end of the task (interspersed by less than ~ 5-s intervals due to ultrasound processing issues). Immediately after the fatigue task, the pre-fatigue shear modulus assessment was repeated. Subsequently, two MVIC trials were performed to verify whether neuromuscular fatigue had occurred. Shear modulus measurements were performed at 20% of MVIC so that results could be compared to similar studies performed on the quadriceps ^13,15^ and because good reliability has been observed at this intensity.^27^.

### Data acquisition and processing

A pedal switch was used to simultaneously start data collection of one ultrasound scanner and the force sensor, while the data acquisition of the second ultrasound scanner was started manually by a local examiner at a similar time when the pedal switch was clicked. The clock time of two ultrasound scanners and the computer where the force was being collected were synchronized before the experiments. As successive SWE clip videos were recorded during the fatiguing task and since the time interval between videos (which was less than 5 s) was not similar between intervals due to processing time, the equipment clocks allowed us to synchronize the SWE data with an error of less than 1 s. We consider this error negligible, as the shear modulus sampling rate was close to 1 Hz.

Shear modulus data were processed using custom Matlab routines (The Mathworks Inc., Natick, MA). Briefly, each clip exported from Aixplorer's software was sequenced into *.jpeg* images. The largest rectangular region of interest (ROI) in the elastogram window was manually selected in each image by avoiding aponeurosis and tissue artifacts (e.g. vessels). Matlab routines excluded grayscale pixels and converted the pixels containing elastogram measurements into elastic moduli values based on the recorded scale. The images where elastograms had less than 50% of area elastogram pixels fulfillment were excluded from analysis, as it increases the measurement error^36^. The elastic moduli values were then averaged to obtain a representative muscle value. It is important to note that the values shown in the elastogram windows of the Aixplorer's ultrasound scanner represent the Young’s modulus of the medium. Thus, values were divided by 3 for the estimation of the muscles’ shear moduli^32^. For the muscle shear modulus observed during the fatigue task, the values were divided into 10 percentiles, the mean value was obtained for each percentile, and the mean values of each percentile were normalized to the first percentile. For the muscle shear modulus observed before and after the fatigue task, the values obtained during a 10-s interval from 5–15 s of each knee flexion trial were averaged, the mean value between the two trials was determined (in both pre- and post-assessments), and then normalized to the values obtained before the fatigue task. Thus, shear modulus data were analyzed using a minimum of 20 data points for each percentile during the fatiguing task (i.e. corresponding to the individual with the minimal time until failure), while a minimum 8 data points (of each trial) were used for the pre- and post- assessments. For the knee flexor MVIC, the highest value observed in each MVIC (before and after the fatigue task) was considered for analysis and used to calculate the fatigue index (i.e. percentage of knee torque decline after the fatigue task). The BFlh/ST shear modulus ratio was also determined for each percentile, and interpreted as a load sharing parameter between these two muscles.

### Statistical analysis

Data analysis was performed using IBM SPSS Statistics (v23, IBM Corporation, Armonk, NY, USA). Normality was confirmed using the Shapiro–Wilk test. In order to examine the consistency of elastogram filling (i.e. % of colored pixels) for each muscle before and after the fatigue task, as well the existence of knee flexor fatigue, a two-way repeated measures ANOVA [day (1, 2 × time (pre, post)] was used. To examine the consistency of elastogram filling of each muscle during the fatigue task, a two-way repeated measures ANOVA [day (1, 2) × time (10, 20, 30, 40, 50, 60, 70, 80, 90, and 100%)] was used.

To examine the reliability and differences in knee flexors’ time to exhaustion between sessions, the intraclass coefficient correlation (ICC_1,1_) and the standard error of measurement (SEM) were determined and a paired t-test was carried out. The ICCs were classified as little if any (0.00–0.25), low (0.26–0.49), moderate (0.50–0.69), high (0.70–0.89), and very high (0.90–1.00)^37^.

The consistency of BFlh and ST absolute shear modulus at the start of the fatigue task (i.e. values of first percentile) was determined using two-way repeated measures ANOVA [day (1, 2) × muscle (BFlh, ST)]. Changes in BFlh, ST, and BFlh/ST shear modulus during the fatigue task in both testing sessions were examined using a two-way repeated measures ANOVA [day (1, 2) × time (10, 20, 30, 40, 50, 60, 70, 80, 90, and 100%)] with simple contrasts (i.e. in comparison to values observed at the first percentile). The immediate effects of the fatigue task on ST, BFlh, and BFlh/ST shear modulus were examined using a two-way repeated measures ANOVA [day (1, 2) × time (pre, post)]. For all ANOVAs, post hoc analyses were performed with Bonferroni’s correction. Significance was set at p < 0.05.

## Results

Data from 1 subject were not considered for analysis since the target muscle region was not correctly captured during the fatigue task. A small percentage of images where elastogram ROIs were filled with less than 50% of coloured pixels during (ST = 0.2%, BFlh = 9.8%) and before/after (ST = 0.1%, BFlh = 11.3%) the fatigue task was also excluded. No significant effects were seen for day, time, or their interaction for elastogram filling during (ST = 96.4 ± 2.1%, BFlh = 74.9 ± 13.9%, p > 0.244; average ROI size: ST = 6.2 cm^2^, BFlh = 5.6  cm^2^) and before/after the fatigue task (ST = 96.5 ± 2.7%, BFlh = 76.3 ± 14.7%, p > 0.074; average ROI size: ST = 6.4  cm^2^, BFlh = 5.7 cm^2^) , suggesting that shear modulus measurements were stable across trials.

A high between-days consistency was observed for the knee flexors’ time to exhaustion [day 1: 443.8 ± 192.5 s; day 2: 474.6 ± 131.7 s; ICC = 0.84 (0.50–0.96); SEM = 43.0 s; p = 0.323]. For the knee flexors’ MVIC, a significant effect was found for time (p = 0.011) but not for day (p = 0.946) or for the day × time interaction (p = 0.189), whereas a small to moderate decrease in knee flexors’ MVIC was found after the fatigue task in both day 1 (− 3.8%; pre = 123.0 ± 26.3 Nm, post = 118.3 Nm, *d* = 0.19) and day 2 (− 7.8%; pre = 125.4 ± 22.8, post = 115.4 ± 17.1, *d* = 0.49).

**Figure 2:**
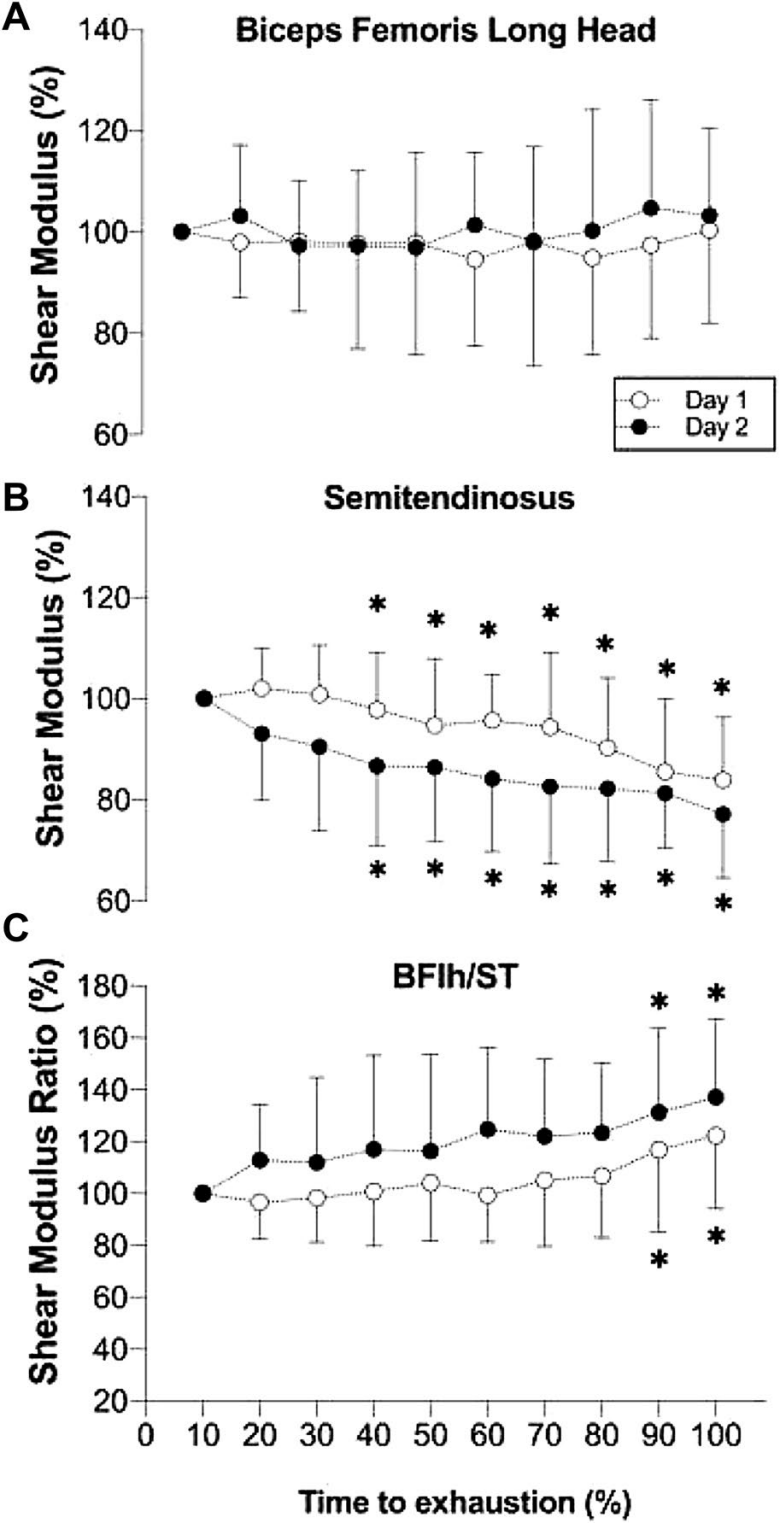
Shear modulus responses for the (**A**) biceps femoris long head (BFlh), (**B**) semitendinosus (ST), and (**C**) BFlh/ST shear modulus ratio, in two separate sessions (day 1 and day 2), during a knee flexors isometric contraction until exhaustion at 20% of maximal voluntary isometric contraction. Data are presented as mean ± standard deviation for each 10% of time to exhaustion. * Significant difference compared to baseline (i.e. initial 10% of the fatigue task time to exhaustion).

At the start of fatigue task, ST (day 1: 61.6 ± 14.9 kPa; day 2: 65.6 ± 13.3 kPa) displayed greater shear modulus than BFlh (day 1: 29.8 ± 7.9 kPa; day 2: 35.0 ± 4.4 kPa; p < 0.001), with no differences between days (p = 0.08). The BFlh, ST, and BFlh/ST relative shear modulus during the fatigue task in both days is shown in Fig. 2. For the BFlh, no significant day (p = 0.494), time (p = 0.770) or day × time interaction (p = 0.684) was found. As for the ST, a significant effect was found for time (p < 0.001), but not for day (p = 0.07) or day × time interaction (p = 0.345). Post-hoc testing revealed a significant decrease in the ST shear modulus from 40% of time to exhaustion (day 1: − 2.2 ± 11.4%; day 2: − 13.3 ± 15.7%, p = 0.027) until the end of the task (day 1: − 16.1 ± 12.4%; day 2: − 22.9 ± 12.6%, p = 0.012). Regarding the BFlh/ST shear modulus ratio, a significant effect was found for time (p < 0.001) but not for day (p = 0.055) or day × time interaction (p = 0.596). Post-hoc testing also revealed that the BFlh/ST shear modulus increased from 90% of time until exhaustion (day 1: + 16.9 ± 31.7%; day 2: + 31.3 ± 32.4%, p = 0.003) until the end of the task (day 1: + 22.3 ± 28.0%; day 2: + 37.2 ± 30.0%, p = 0.001).

Regarding the immediate effects of the knee flexion task on the shear modulus, no significant day (p = 0.944), time (day 1: + 2.7 ± 12.9%; day 2: + 2.2 ± 17.3%; p = 0.406), or day × time interaction (p = 0.944) effect was seen for the BFlh. For the ST, a significant time (day 1: − 11.8 ± 12.4%; day 2: − 17.8 ± 14.2%, p < 0.001), but not day (p = 0.325) or day × time interaction (p = 0.325) effect was found. Finally, for the BFlh/ST ratio, a significant time (day 1: + 18.4 ± 21.3%; day 2: + 28.1 ± 32.5%, p < 0.001), but not day (p = 0.479) or day × time interaction (p = 0.479) effect was found.

## Discussion

The present study examined the BFlh and ST active stiffness during and immediately after a knee flexion fatigue protocol. To the best of our knowledge, this is the first study providing evidence of changes in the hamstring active stiffness pattern during and immediately after a submaximal isometric contraction fatiguing task in a consistent manner across two sessions. Specifically, we noted that the ST active stiffness began decreasing at 40% of the time until exhaustion. Additionally, the ST active stiffness was still decreased immediately after the task. Conversely, no significant changes were observed in the BFlh active stiffness, either during or after the task.

Few studies have examined the effects of fatiguing exercises on localized muscle active stiffness through the use of SWE^13–16^, rather than through global joint measures. All these studies have examined the effects of knee extensor fatiguing protocols on quadriceps active stiffness. However, conflicting results from these studies suggest a dependency of the fatiguing protocol used. For instance, Bouillard et al. have observed inter-individual variability in the active stiffness of the quadriceps muscles during a knee extensor isometric contraction at 20% of MVIC performed until exhaustion, where a considerable decrease of knee extensor MVIC was noted^13,15^. On the other hand, a *vastus lateralis* active stiffness decrease was noted when using a fatiguing protocol with an intermittent knee extensor isometric contractions with higher contraction intensity (i.e. 60–100% of MVIC), which suggests that the variability in the inter-individual load sharing response to fatigue depends on the fatiguing protocol^14,16^. In the present study, we used a similar protocol to Bouillard et al. on the knee flexor muscles^13,15^. We observed a consistent ST and BFlh active stiffness pattern response among individuals in two replicated sessions with a 7-day separation. This indicates that the type of load sharing response with fatigue is not only dependent on the fatiguing protocol, but also dependent on the type of muscle groups involved in the fatigue task.

Knee flexion requires a higher number of muscular actuators with distinct tendon insertions compared to knee extensor muscles, which share a common tendon. The higher number of actuators increases the possibilities of muscle combinations to generate a given joint torque. As redundancy is higher for the knee flexor muscle group, it would be plausible to expect a variable inter-individual response between the ST and BFlh with fatigue. However, this was not the case. One possibility to explain this phenomenon could be the nature of synergistic active stiffness in a non-fatigued state. For instance, in knee extensor isometric contraction at low intensities in a non-fatigued state, the active stiffness of the vastus lateralis, vastus medialis, and rectus femoris are not consistent between individuals^13,15^. It should be noted that at lower intensities, the *vastus intermedius* appear to have a higher relative contribution to knee extension torque^38^, as it has a greater proportion of slow-twitch fibers compared to the other quadriceps muscles^39,40^. Also, the proportion of slow/fast twitch fibers seems to have high inter-individual variability in certain superficial quadriceps muscles as the *vastus lateralis*^17^. On the other hand, the hamstring active stiffness in a non-fatigued condition during the knee flexor task with similar intensity is consistent among individuals and between days, with a greater ST active stiffness compared to BFlh^27^. Such hamstring load sharing pattern has also been noted at the metabolic and neural levels^24,26^. Thus, we hypothesize that an active stiffness decrement within a given synergistic muscle depends on whether that muscle is consistently required with a greater (relative) work. Previous research has reported a different ST-BFlh recruitment pattern with hip extensor exercises.^26^ Thus, it could be interesting to examine in future research if the ST-BFlh active stiffness pattern response to a fatigue stimulus differs between a hip extensor vs. knee flexor protocol, where the demands imposed on the hamstring muscles are different.

Moreover, two additional aspects could explain the changes in the BFlh-ST active stiffness pattern: muscle fiber composition and temperature. In terms of muscle fiber composition, early research has suggested that hamstring muscles are mainly composed by glycolytic fibers (i.e. type IIx/b fibers)^21^. However, other studies have evidenced that this might not be the case among all hamstring heads^22,40^. In particular, the BFlh seems to have a considerable high number of type I/IIa fibers^22,41^, although the percentage of glycolytic fibers has the potential to increase^42^. Conversely, the ST has been reported to have a considerably higher proportion of fast-twitch fibers^31^. Thus, we contend that ST and BFlh may have a consistently different proportion of slow/fast twitch fibers, making the ST a less fatigue-resistant muscle. Considering that the muscle force-stiffness relationship is unaffected over the course of the fatigue task^11,12,43^, a different fiber composition between the ST and BFlh could explain the early decrease in ST active stiffness during fatigue installation while the BFlh active stiffness is unaltered. Future research should examine this hypothesis. Regarding muscle temperature, it is known that increased muscle temperature derived from muscle contraction decreases muscle stiffness^11,43,44^. Unfortunately, we did not measure muscle temperature in this study. Nonetheless, we do not know whether the exercise-induced increase in intramuscular temperature differs between synergistic and neighboring muscles. We strongly believe it is very unlikely that temperature change could have influenced the ST active stiffness decrease without affecting BFlh.

The present findings might open perspectives from a motor control and clinical perspective. Considering that the muscle force-stiffness relationship was unaffected (e.g. by temperature)^11,43^, changes in active stiffness is indicative of altered muscular force derived from contractile machinery^9,12,44^. If such premise is correct, and assuming that an equal change in shear modulus between ST and BFlh muscles reflects a similar alteration in terms of force, we wonder if this could affect knee stability through an altered load imbalance between medial and lateral hamstring, favouring injury occurrence^45,46^. For instance, a lower ST active stiffness combined with lower activation may favour exaggerated knee valgus and tibia external rotation, in actions as drop jumps and side cutting, a well-known major risk factor and injury mechanism for the tear of the anterior cruciate ligament^47,48^. It should be noted that other muscles (i.e. semimembranosus and biceps femoris short head) that could influence the medial/lateral hamstring imbalance were not assessed in this study. It is desirable that a future study contemplates these muscles that generate a considerable contribution to the forces manifested in the knee. Moreover, an increased BFlh/ST active stiffness ratio is also suggestive of a load sharing pattern favouring BFlh overload and ST underload. This workload pattern has been associated to hamstring strain injury^25,29^. It is also important to note that ST active stiffness decrease was noted from 40% of time to exhaustion, with minimal knee flexor MVIC decrease (i.e. − 3.8 to − 7.8%) immediately after the fatiguing task, that persisted ~ 5 min after the fatigue task. Previous studies on the quadriceps have reported higher indexes of fatigue with changes in synergistic load sharing^14,16^. This means that the hamstring active stiffness pattern may change with minimal fatigue-induced knee flexors maximal strength loss, contrary to what is seen in the quadriceps. It would be interesting to examine if the hamstring active stiffness pattern alteration is higher with greater levels of fatigue. Future research is warranted to examine whether an altered hamstring active stiffness pattern is associated with injury occurrence.

This study presents some limitations. (1) Shear modulus measurements were performed at a single muscle site and length; we do not exclude the (potential) existence of shear modulus variability within muscle sites and at different lengths. (2) We have not measured the shear modulus of the muscles at rest; thus, it is not possible to determine whether changes occurred in muscles’ passive properties. (3) While the focus of this research was the BFlh and ST, other muscles also contribute to knee flexion (e.g. gastrocnemius) and tibial rotation (e.g. semimembranosus) and can be a part of the local muscle strategy to maintain task performance. We have not assessed these muscles which can play a role in attenuating muscle fatigue. (4) A small percentage of the elastogram windows (i.e. < 11.3%) had missing elastography values during the fatigue task. As this study examined within-task changes and no significant differences were found across testing moments in both sessions, we assume the unfilled windows did not not induce bias in the study findings. (5) The study findings are specific to the contraction type, mobilized joint, muscle length, and tested population (e.g. male vs. female) we assume results may differ depending on these factors. (6) While recent research has reported that the calculation of shear wave group velocity overestimates the tissue shear elastic modulus^35^, we think the study findings are not biased as within-subject comparisons over time were analyzed.

In conclusion, this study showed for the first time that a knee flexor isometric contraction at 20% of MVIC until exhaustion alters the hamstring muscles active stiffness pattern, with inter-individual consistency between two testing sessions. Semitendinosus active stiffness decreased from approximately half the time of the fatigue task, without changes in the BFlh. Our approach provides a potentially more accurate tool to analyze load sharing among individual muscles under normal and pathological situations subjected to the fatiguing tasks. This opens perspectives in various fields, such as motor control, injury prevention, rehabilitation, and biomechanics.
